# Probabilistic MRI Brain Anatomical Atlases Based on 1,000 Chinese Subjects

**DOI:** 10.1371/journal.pone.0050939

**Published:** 2013-01-02

**Authors:** Wang Xing, Chen Nan, Zuo ZhenTao, Xue Rong, Jing Luo, Yan Zhuo, Shen DingGang, Li KunCheng

**Affiliations:** 1 Department of Radiology, Xuanwu Hospital, Capital Medical University, Beijing, China; 2 State Key Laboratory of Brain and Cognitive Science, Beijing MRI Center for Brain Research, Institute of Biophysics, Chinese Academy of Sciences, Beijing, China; 3 Department of Radiology and Biomedical Research Imaging Center, The University of North Carolina at Chapel Hill (UNC-CH), Chapel Hill, North Carolina; Beijing Normal University, Beijing, China

## Abstract

Brain atlases are designed to provide a standard reference coordinate system of the brain for neuroscience research. Existing human brain atlases are widely used to provide anatomical references and information regarding structural characteristics of the brain. The majority of them, however, are derived from one paticipant or small samples of the Western population. This poses a limitation for scientific studies on Eastern subjects. In this study, 10 new Chinese brain atlases for different ages and genders were constructed using MR anatomical images based on HAMMER (Hierarchical Attribute Matching Mechanism for Elastic Registration). A total of 1,000 Chinese volunteers ranging from 18 to 70 years old participated in this study. These population-specific brain atlases represent the basic structural characteristics of the Chinese population. They may be utilized for basic neuroscience studies and clinical diagnosis, including evaluation of neurological and neuropsychiatric disorders, in Chinese patients and those from other Eastern countries.

## Introduction

A greater understanding of the relationship between the human brain anatomical structure and function is important in neuroscience research [1]. In recent years, the development of human brain atlases has provided a standard platform for the accurate assessment of brain function and correlation to various microscopic and macroscopic anatomical structures. The Talairach and Tournoux atlas is the most commonly used human brain template, which was developed based on postmortem sections of a 60-year-old French female, with the slice space ranging from 3 to 4 mm [2]–[4]. Brodmann's map published in 1909 divides the cerebral cortex into 43 regions based on cytoarchitectonic subdivisions [5]–[7], and remains widely used as a neuroanatomical approach to examine brain structural-functional correlations. Standard MNI brain templates based on several hundred individual MRI scans are widely used as average brain templates [3]. They are a series of atlases made of different methods and parameters. These atlases work for different analysis and are able to be free downloaded on the MNI website. Furthermore, the International Consortium of Brain Mapping (ICBM) has created a series of brain templates to be used as standards atlases [1], [4]. In Asia, a Korean brain template based on 78 Korean normal volunteers was developed in 2005 [8]. This was an early eastern template that could represent the brain characters of Asian population. More recently, a Chinese brain atlas was also constructed from MRI scans of 56 Chinese male subjects, and the results were compared to an age-matched cohort of 35 Caucasian males [9].

The human brain is highly variable between individuals and phenotypically different groups (e.g., age, gender and race). Standard brain templates are therefore crucial for reducing subject anatomical variation, providing normalized anatomical references for individual- or population-based assessment of brain function and structure and for the diagnosis of neurological diseases. Although brain templates and brain atlases have been widely used in fMRI, clinical medicine, and other neuroscience research fields, they are not strictly designed according to different ages, genders or other factors. Additionally, the anatomical differences between Western and Eastern populations provide the greatest variation, with fundamental genetic and environmental disparities resulting in overall and regional differences in brain shape, size and volume [8]–[9]. Current Western templates are also often designed from small brain samples that may not always represent group differences in gender and age. Thus, the use of popular templates created specifically from Western human brain samples may result in the mislocalization of activated brain regions measured with functional MRI [10]–[11], and in positional mismatches during image-guided stereotactic neurosurgery for Chinese patients. As such, it is critical to develop Chinese brain templates for neuroscience research. In the present study, a set of group-wise anatomical Chinese brain probabilistic anatomical atlases of different ages and genders were established using a deformable brain registration method, HAMMER [12].

In recent years there have also been a number of probabilistic brain atlases for pathological cohorts (e.g Parkinson's disease, Alzheimer's disease, and so on). For example, template-based brain MRI image segmentation of deep brain structures (e.g subthalamic nucleus) of patients with Parkinson's disease may be preferable for MRI image analysis in these patients [13]. Probabilistic maps have also been created according to the requirement of visualization of neurosurgery and functional brain imaging [14]. A well-established probabilistic segmentation model with anatomical tissue priors based on data from the Alzheimer's disease Neuroimaging Initiative (ADNI) enabled a new platform for the probabilistic brain atlases [15] template-based techniques. Various analytic methods of brain structures were also developed to help brain disease research in these years. Therefore, construction of high-resolution MRI-based brain structure atlases using a large number of 3D MRI images will be highly useful in neurosurgery as well as anatomical and functional studies of the human brain.

## Materials and Methods

### Subjects

A total of 1,312 normal subjects ranging in age from 18 and 70 were recruited from 15 hospitals in China (these data were collected through the CD-ROM or hard disk from each hospital) for about 2 years. The participating hospitals are Affiliated Hospitals of the key universities in China. Each subject underwent a medical examination to exclude subjects with a lifetime history of any neurological, psychiatric, or significant medical illnesses as well as patients with a past history of substance abuse. All subjects were subdivided into five age groups (18–30, 31–40, 41–50, 51–60 and 61–70 years). Each age group was further divided into two gender groups. Ten groups of data were used to establish the templates. The atlases were established over the course of around 1 year. This study was approved by our institutional review committee (the ethics committee of Xuan Wu hospital, Capital Medical Unversity), which met the guidelines of our responsible governmental agency. Written consent was obtained from each volunteer. This study was registered in the Clinical Trial Register (Registration Number: ChiCTR-RNC-00000128).

### Image acquisition

All volunteers underwent a whole brain scan with T1-weighted, T2-weighted and 3D T1-weighted MP-RAGE sequences using 1.5T MR scanners (Sonata Siemens Medical Systems, Erlangen, Germany). The parameters were: flip angle = 15°, TR/TE/TI = 2000/4–4.5/1100 ms with 192 slices, slice thickness = 1 mm (there is no inter-slice thickness), image field of view = 256×256 mm2, and in-plane image resolution = 256×256, leading to an isotropic voxel size of 1×1×1 mm3. The imaging time was 13 min per 3D data set. Prior to further analysis, all MR images were exported in a conventional format and inspected by experienced radiologists in each hospital. Subjects with any abnormalities including brain tumors, infarctions or white matter degeneration (i.e., diameter greater than 5 mm) were excluded from the study. There abnormalities were identified using T1-weighted and T2-weighted MR sequences. The researchers developed a guidance manual and then a unified volunteer inclusion and exclusion criteria and MRI scan parameters are provided to ensure the uniformity of data. Out of the 1,312 images, we chose 1,000 images that met the requirements and then subdivided them into ten groups (100 images per group).

### Data preprocessing

[1] Prior to using the HAMMER algorithm to align brain images and generate brain templates [16], raw MR images were preprocessed using the following steps:

- Format conversion using the MRIConvert tool. Raw data exported in DICOM-format from the Siemens Workstation was first transformed into Analyze format with header information.
- Brain images were reoriented to AC-PC (anterior commissure-posterior commissure) position and further aligned to the same brain position by MIPAV (Medical Image Processing, Analysis, and Visualization, NIH, USA).
- For skull stripping and tissue segmentation of MR brain images, the BET2 and FAST algorithms, respectively, were used to obtain gray matter (GM), white matter (WM) and ventricles (VN) via the FSL package (FMRIB Analysis Group, Oxford, UK). The cerebellum was retained to keep the brain intact during the HAMMER-based brain normalization procedure.
- After skull stripping and tissue segmentation, each brain tissue region was assigned a specific value using MIPAV according to the requirement of the HAMMER algorithm, i.e., 250 for WM, 150 for GM, 50 for VN and 10 for CSF.

### Selection of an optimal target brain image

Before data processing, all of the brain volumes were measured by calculating the total number of voxel in each image data using matlab (MATLAB, the Math Works Inc, Natick, and Mass). In each age and gender group, a brain image with a volume that much close to the mean volume, intact brain structures and global brain symmetry was selected to serve as an initial template. The template for each age group was selected by two experienced radiologists and two imaging specialists who used their expertise and followed the morphological measurements of head compartments volumetry [17]–[18]. During this procedure, three cross-sectional views (axial, coronal and sagittal views) of the brain image were displayed using MIPAV software. With reorientation of AC-PC in the preprocessing step, the length, width and height of each brain were able to be directly measured. These measurements were used to select 40 subjects with the lowest deviations from the mean image to serve as candidates for the initial optimal target brain images. Together, the experienced radiologists and imaging specialists then selected 20 optimal brain images to serve as the initial templates for the 10 age and gender groups. Two optimal brain images were chosen as the initial template in each group. Then researchers repeated the registration processing twice, once for each optimal brain image.

### Processing

To align each image in the respective age group to a selected template, we used a deformable registration algorithm named HAMMER. HAMMER uses two novel strategies to improve registration performance [15]. First, an attribute vector (i.e., a set of geometric moment invariants (GMIs)) was defined for each voxel in the image to reflect the underlying structural information about the local image around that voxel. The use of an attribute vector can help distinguish between different parts of the image and can establish the anatomical relationship between the two images under registration, thus reducing the possibility of being trapped by the local minima. Second, a hierarchical registration strategy was used for progressively registering images. In particular, it used the voxels with distinctive attribute vectors to guide the initial registration. Other voxels simply followed the registration of the distinctive voxels through interpolation of the deformation field. As registration progressed, more and more voxels with less distinctive attribute vectors were included to join the registration of the images. This further refined the registration results. By using these two novel strategies, the HAMMER registration algorithm was able to register images with relatively high accuracy and sharpened structural information.

With selection of the initial template, the first registration process was done. All other brain image samples (N-1) within the same age group were registered to this initial template. The registration was done based on all image data within one group. After the first registration process, the deformation fields estimated for all brain image samples were averaged to generate an average deformation field. This average was then used to transform the current template to a location for generating a warped template. This was done by hierarchically refining the displacement fields using local and global affine transformations that were calculated from the deformations in the template driving voxel. After the first registration process, an individual brain image sample that was the closest to this warped template was then selected as a new template and the second registration procedure was repeated, as stated above, based on all image data within a group. This procedure was conducted until the whole process converged. By doing so, all brain images were ultimately normalized to their geometric center, and further averaged to generate an average brain atlas. Data processing was repeated twice by two group members in the same computational environment (XuanWu group and Institute of Biophysics group).

## Results

**Figure 1:**
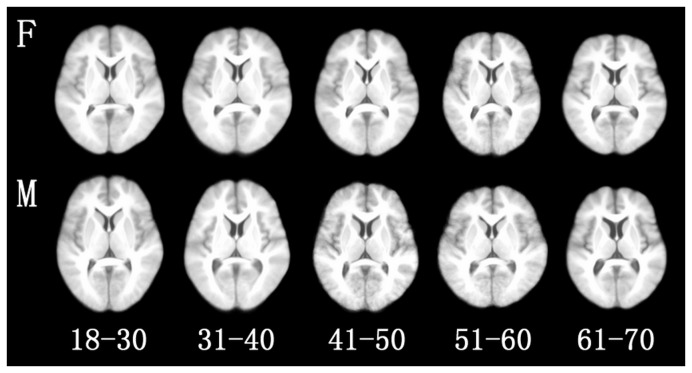
Axial views of 10 Chinese brain atlases of different age and gender groups. The atlases of different ages are showed from left to right. F, female atlases. M, male atlases.

Based on the procedures described above, ten IMA formatted atlases have been created and will soon be made available online for free downloads. The axial views of the 10 probabilistic atlases of human Chinese brains are shown in Figure 1. The atlases of different ages are displayed from left to right (female atlases on the top line, male atlases on the second line).

The size and volume of each atlas were also measured using matlab (MATLAB, the Math Works Inc, Natick, and Mass) programs by calculating the total number of template pixels (Table 1). In our opinions, the total number of pixels in one atlas represents its volume.

**Table 1:** Parameter measurements of 10 Chinese brain atlases of different ages and gender groups.

Group	F					M				
	1	2	3	4	5	1	2	3	4	5
Volume(cm3)	1660.023	1546.889	1504.574	1439.555	1385.483	1755.521	1681.524	1621.92	1504.884	1484.361

**Figure 2:**
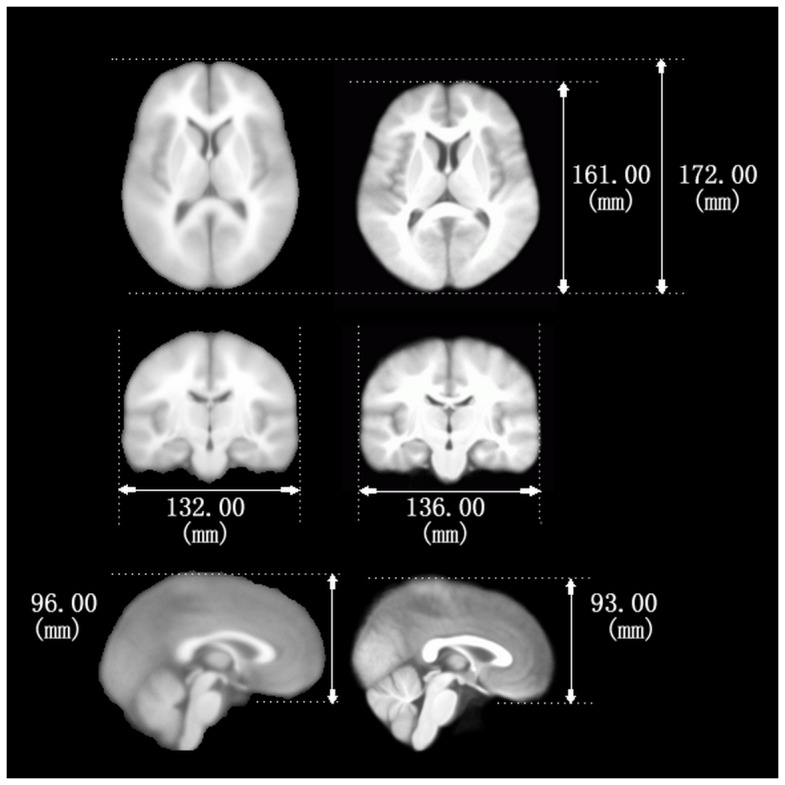
Comparison between the Chinese atlas and the MNI atlas. Three cross-sectional figures of the MNI alas and the Chinese atlas are shown on the left and the right, respectively. Measurement parameters are also marked on the map.

Additionally, the atlas of Group 1 was rigidly aligned with the MNI template (average age, 23.4±4.1 years) and the brain size and volume were compared. Differences in global features, including shape and size, of the two atlases, are shown in Figure 2.

Comparatively, the Chinese brain templates are generally smaller in length and height than the MNI template of the Western society, while the width/length ratio for the average Chinese brain is larger than the MNI brain template (Figure 2).

**Figure 3:**
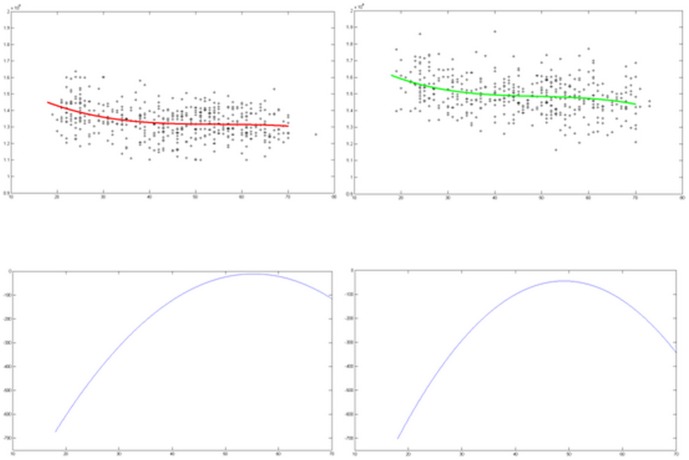
A) Brain volume changes from 18 to 70 years. X-axis, age in years. Y-axis, volume (mm^3^). Left, female. Right, male. The curve fitting equation was: 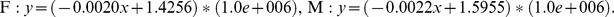 B) The curves represent the mean trajectory of the two equations.

After generation of group atlases, parameter measurement and analysis of each brain from the different groups was performed. It is well established that the human brain changes with age [19]–[21]. Therefore, the volumes of all brains based on raw data were further examined to assess age-related changes. A trend of decreasing brain volume with increasing age was found (see Figure 3). The brain volumes of males were significantly larger than that of females (showed in panel A). The ratio of brain volume changes according to ages was measure by calculating the slope of the line in panel A (Fig. 3 panel B). The reduction in the ratio of brain volume continued to increase until approximately 55 years in females and 50 years in males (showed in panel B). We conducted a statistical analysis using a paired t-test (SPSS software, version 14.0) to perform a group analysis. There was a significant difference between females and males in each group (18–30, 31–40, 41–50, 51–60 and 61–70 years) p<0.01).

## Discussion

In this study, 10 new Chinese brain atlases were constructed using MR images based on 1,312 Chinese volunteers' ages 18 to 70 years old. Differences of brain patterns were identified with age and gender. These series of atlases may prove to be useful standard templates that represent brain characteristics of the Chinese population of different ages and genders.

To develop atlases with good spatial resolution and clear structural information, a reliable and accurate non-rigid registration algorithm termed HAMMER was employed. HAMMER is a deformable registration algorithm of medical images that exhibits high accuracy in superposition that hierarchically warps and averages brain images from different subjects [15], [22] (Fig. 1). HAMMER is commonly used for analyzing images from people with brain diseases [15], [23]. The sharp boundaries of the cortices, ventricles, white matter and grey matter regions observed in the 10 average brain templates provide further support for the benefits of this algorithm.

So far Talairach and Tournoux atlas may be the most popular used human brain template. The disadvantage is that the origin of this atlas was based on postmortem sections of an old French woman. It is an old and inactive sample. As far as the MNI templates, these atlases are widely used in many kinds of fMRI analysis. The comparison was also done in this study and the results showed there indeed existed differences between MNI atlas and Chinese brain atlases. The advantage of Brodmann's map is the division of 43 regions of the cerebral cortex based on cytoarchitectonic subdivisions. This will be a future work for the ten Chinese brain atlases to divide into more accurate anatomical structural regions. Compared with the other two Asian templates, the advantages of our ten Chinese Atlases cover a larger number of samples and age-gender classification.

Although it is well known that functional differences exist between brain regions of Eastern and Western people [24], [25], little is known regarding the underlying structural differences [26], [27]. In this study, Chinese brains were typically smaller in length and height than Western brains based on both the atlases and the raw data. Furthermore, volumetric brain parameters were larger in males than females, and an age-related decline in brain volume was observed (see Fig. 3).

One phenomenon discovered in this study was that male brain volumes were larger than female brain volumes (Fig. 3 panel A). This was examined by calculating every brain volume in each participant from 18 to 70 years. Female brain volumes continue to increase from 18 to 55 years and then rapidly decline after 55 years. Male brain volumes continue to increase from 18 to 50 years and then decline after 50 years (Fig. 3 panel B). In our study, although the brain volumes of males are larger than those of females, the onset of the decay of the brain volume is earlier in males. A similar age-related deterioration of brain volume has been previously reported [21], [28]–[33].

Despite these 10 atlases of different age and genders, further population-specific, group-specific and disease-specific atlases are required for advance neuroimaging research. A potential limitation of the atlases in our study is that they are static and do not show dynamic brain changes with time, compared to dynamic 4D probabilistic atlases. Our atlases were constructed from 1.5T MRI scanner data, while future template construction should include 3.0T MRI data. Finally, limitations in existing registration methods may still cause loss of detailed information in the templates. Thus, more optimized data collection and image processing methods are required.

In conclusion, 10 atlases representing basic brain structural characteristics of the Asian population were developed. We suggest that these 10 Chinese brain probabilistic atlases would provide a more suitable basis for Chinese neuroscience studies and clinical diagnosis than the widely used Western brain atlases, owing to the structural differences between Chinese and Western populations. We will continue to collect MRI data on different sub-populations including people with different race and diseases to further optimize these Chinese brain atlases.
