# Correction: Probabilistic MRI Brain Anatomical Atlases Based on 1,000 Chinese Subjects

**DOI:** 10.1371/annotation/c4d2aff9-0c5c-4ebb-b1ed-efa69fc84d78

**Published:** 2013-04-22

**Authors:** Wang Xing, Chen Nan, Zuo ZhenTao, Xue Rong, Jing Luo, Yan Zhuo, Shen DingGang, Li KunCheng

There was an error in the funding information. The correct funding statement is:

This work was supported by the Ministry of Science and Technology of China grants (2006AA02z391, 2005CB522800, 2004CB318101), National Natural Science Foundation of China grants (30621004, 81271556), and the Knowledge Innovation Program of the Chinese Academy of Sciences. The funders had no role in study design, data collection and analysis, decision to publish, or preparation of the manuscript.

